# The Medical Orders for Scope of Treatment (MOST) form completion: a retrospective study

**DOI:** 10.1186/s12913-022-08542-w

**Published:** 2022-09-22

**Authors:** Anastasia A. Mallidou, Coby Tschanz, Elisabeth Antifeau, Kyoung Young Lee, Jenipher Kayuni Mtambo, Holly Heckl

**Affiliations:** 1 School of Nursing, University of Victoria, B236 – HSD Building, 3800 Finnerty (Ring) Road, Victoria, BC V8P 5C2 Canada; 2 Palliative Care and End of Life Services, Interior Health, Vancouver, VIC Canada; 3 Vancouver Coastal Health, Vancouver, VIC Canada; 4 Dr. Peter AIDS Foundation, Vancouver, Canada

**Keywords:** Advance care planning, Medical Orders for Scope of Treatment (MOST) form, Hospital, Nurse practitioner, Retrospective study, MOST form completion, Health system quality and safety

## Abstract

**Background:**

Advance care planning (ACP) involves discussions about patient and families’ wishes and preferences for future healthcare respecting autonomy, improving quality of care, and reducing overtreatment. The Medical Orders for Scope of Treatment (MOST) form records person preferred level and types of treatment and intervention.

**Purpose:**

To examine the MOST form use in inpatient units within a British Columbia (Canada) hospital, estimate and compare its completion rate, and inform health policies for continuous, quality and individualized patient care.

**Methods:**

About 5,000 patients admitted to the participating tertiary acute care hospital during October 2020. Data from 780 eligible participants in medical, surgical, or psychiatry unit were analyzed with descriptive statistics, the chi-square test for group comparisons, and logistic regression to assess predictors of the MOST form completion.

**Results:**

Participants’ (54% men) age ranged from 20–97 years (mean = 59.53, SD = 19.54). Mainly physicians (99.1%) completed the MOST form for about 60% of them. A statistically significant difference of MOST completion found among the units [Pearson χ^2^_(df=2, *n*=780)_ = 79.53, *p* < .001, φ = .319]. Multivariate logistic regression analysis demonstrated that age (OR = 1.05, 95% CI 1.04 to 1.06) and unit admission (OR = .60, 95% CI 0.36 to 0.99 in psychiatry; and OR = .21, 95% CI 0.14 to 0.31 in surgery) were independently associated with the MOST form completion.

**Conclusion:**

Our findings demonstrate a need for consistent and broad completion of the MOST form across all jurisdictions using, desirably, advanced electronic systems. Healthcare providers need to raise awareness of the MOST completion benefits and be prepared to discuss topics relevant to end-of-life. Further research is required on the MOST form completion.

## Background

Advance care planning (ACP) is a complex future-planning activity that coordinates and supports progressive discussions with multiple stakeholders (e.g., patients, family, friends, clinicians, policy-makers) about personal preferences, values and wishes for future healthcare. ACP is an ongoing process that is done “early and often” (i.e., reviewed frequently) as the best standard to achieve goal-concordant care [1, 2]. This process may result in decisions and the creation of documents, depending on local legislation requirements, to explore healthcare options and support substitute decision-making. The underlying goals of ACP include respecting individual autonomy, improving quality of care, strengthening relationships, preparing for end-of-life, and reducing overtreatment [3], as well as improving concordance between wishes of patient and care received, and between patient and surrogate wishes, higher incidence of preferred place of death, and reducing use of healthcare resources (e.g., hospitalizations, unwanted life-sustaining procedures) [4]. Enhanced attention to and skills in facilitating ACP conversations and communicating decisions is a health professional priority for improving healthcare, especially for people living with advancing life-limiting illness [5]. ACP is related to but differentiated from **Goals-of-Care (GoC)** that refers to discussion and/or record of current wishes and preferences for personal and healthcare decisions that are decided by the person (or substitute decision-maker) in the moment. GoC defines a person’s wishes for treatment in one’s current situation that may change over time; whereas ACP identifies wishes and preferences for possible future health events. Healthcare that aligns with known GoC is called “goal concordant care” [6]. Discordant care can be viewed as a medical error [7].

The **Medical Orders for Scope of Treatment (MOST)** form is a document to record individual current GoC [8] specific to person wishes, choices and preferences for healthcare and guide decision-making in the event of a change of condition that requires decision-making about intervention [9, 10]. The MOST form completion is a result of ACP and GoC conversations in the current context of serious and advancing life-limiting illness and specifies a designation that outlines preferred level and types of treatment [11, 12]. Notably, there are many different types of life-sustaining documentation worldwide (i.e., MOST-type documents) with different and various names, but all record individual wishes and preferences. For example, the Physician or Portable Orders for Life-Sustaining Treatment (POLST), the Medical Orders for Life Sustaining Treatment (MOLST), and the Treatment Escalation/Limitation Plan (TELP) forms. The MOST-type documents (not interchangeable with advance directives) offer more detail about care preferences than a code status designation [13].

### MOST in Canada & British Columbia

In Canada, a 5-step ACP process (i.e., thinking, learning, deciding, talking, recording) was designed to guide persons through legal documents that empower substitute decision-makers to honour their wishes and preferences for future healthcare, should the person be unable to make health decisions for themselves [14, 15]. However, Canadian provinces and territories have different legislation and nomenclature for both the substitute decision-maker and the legal documents authorizing a proxy decision-maker [16, 17]. In addition, while similar MOST forms are used across all five BC health authorities and in other Canadian provinces (e.g., Manitoba), these forms are not identical. When individuals are transferred out of the region, a different MOST format or directive is necessary. Currently, the BC Centre for Palliative Care is exploring standardization of the MOST form for shared use across BC to support provincial-wide continuity of patient care. MOST forms in use list six designations ranging from comfort care to critical care interventions such as CPR and intubation [18]. A physician or nurse practitioner (NP) is responsible for conducting and documenting the MOST form upon individual admission in consultation with the person admitted in the hospital or their designated substitute decision-maker, regardless of where they are located (e.g., home, hospital, long-term care facility). If a MOST form has been completed previously, it must be reviewed within 24 h of re-admission. In the absence of MOST, individuals are default to conservative care including CPR and full medical interventions to address life-threatening events.

### ACP benefits & gaps in knowledge

Individuals, their families, and the health system may benefit of ACP. These potential benefits include honouring autonomy and dignity, increasing alignment between care preferences and delivery (“goal-concordant care”) as well as efficiency of care, improving team communication for continuity of care, avoiding transfer to hospital, and reducing length of stay and expense of unwanted diagnostics and treatments. However, evidence shows that individual healthcare preferences are not consistently explored, recorded or reviewed prior, during, or post hospital admissions [19, 20]. Therefore, we aimed to examine the MOST form completion in BC.

### Purpose & objectives

The purpose of this study was to engage various stakeholders (e.g., healthcare practitioners, students, researchers) in examining the MOST form and its use within an acute care hospital in a specific BC health authority, and to inform health policies to support individuals, families and healthcare providers to share open discussions about ACP and, ultimately, inform continuous, quality and individualized patient care. Our main objectives were to a) estimate the completion rate of the MOST form in three inpatient units or specified beds (i.e., medical, surgical, and mental health/psychiatry); b) compare the completion rate of the MOST form among patient demographics (e.g., age, sex) and units; and c) estimate predictors of the MOST form completion.

## Methods

### Design

This research project is a retrospective, non-experimental study.

### Sample & eligibility criteria

The eligibility criteria of our target population include age, admission beds/unit, and admission date. Therefore, our accessible population and sample consists of adults older than 19 years of age who were admitted to medical, surgical, or mental health (psychiatry) beds/units during a one-month period (from the 1^st^ to 31^st^ day of October 2020 inclusive) in a tertiary acute care hospital within one BC health authority, regardless of other demographic characteristics or diagnoses. Persons treated in other units such as outpatient clinics or surgical daycare were excluded. A total of 780 eligible participants were included in the study.

### Data collection process

We collected anonymized personal information from charts and other clinical documents (e.g., electronic health records) with assistance from the information technology (IT) personnel in the participating BC health authority. Specifically, the data were collected and provided from the health authority to the research team in anonymized format. Data were extracted from the health authority databases according to a detailed spreadsheet that we had provided. The research team neither had nor retained any personal identifiers. Instead, a unique number was assigned by the researchers as the identifier for each participant (e.g., P1, P2, P3), which was not related with any patient identification number provided by the health authority or hospital. The data were originated in the health authority and transferred to the research team using a secure share site location. The health authority uploaded the spreadsheet with the requested data and provided access to the principal investigator, who was able to easily and effectively download, open and save the file in a password-protected and encrypted server. The collected data included demographic characteristics (e.g., age, sex); completion of the MOST form (i.e., yes, no); health professional who completed the form (i.e., physician, nurse practitioner, resident, support worker, inactive physician); admission unit (i.e., medical, surgical, psychiatry); and dates of admission and of the MOST form completion.

### Data analysis

To address the study objectives, we analyzed the collected data using descriptive (e.g., frequency, mean, median, range) and inferential (e.g., chi-squared, logistic regression) statistics with the IBM SPSS Statistics version 26.0 software. In particular, we assessed the completion rate of the MOST form using descriptive statistics; and we compared the completion rate among various groups (e.g., patient demographics, units) by using the Pearson chi-squared test (non-parametric test for differences between two independent groups with outcome variable at nominal level). Finally, we assessed potential predictors of the MOST form completion by using logistic regression. The level of significance has been set at 0.05 for all statistical analyses.

## Results

### Descriptive statistics

**Fig. 1:**
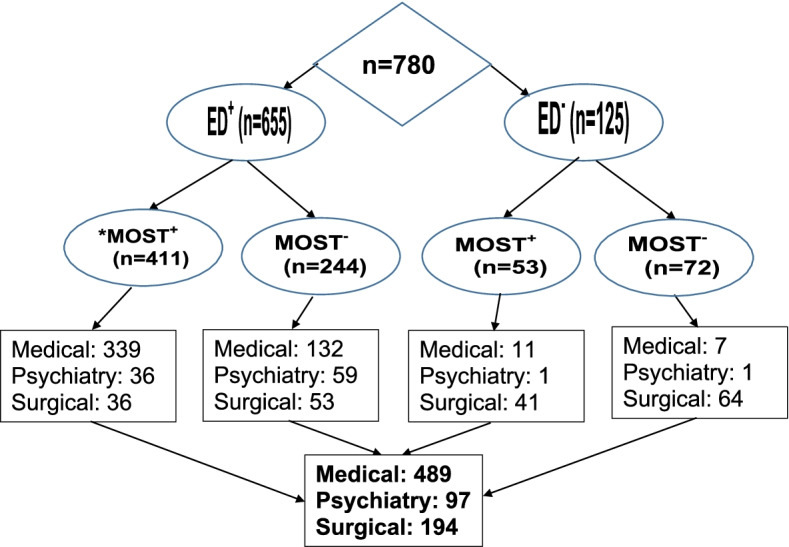
MOST Completion

During October 2020, in total 5,050 persons of any age, sex or diagnosis were admitted in the participating hospital. From these admissions, 780 eligible participants’ data were analyzed in this study. As indicated in Table 1, the participants (*n* = 780; 15.46% of all admissions) hospitalized in medical (*n* = 489; 62.61%), psychiatry (*n* = 97; 12.42%) or surgical (194; 24.84%) unit/beds with an admission rate from 10 to 38 patients daily. In our study, the participants’ age ranged from 20 to 97 years with a mean of 59.53 years (SD = 19.54). There were slightly more males (*n* = 465; 54%) than females (*n* = 361; 46%). Out of 780 admissions, the majority of participants (*n* = 655) were admitted through the Emergency Department (ED) in medical (*n* = 471; 71.9%), psychiatry (*n* = 95; 14.5%), or in surgical (*n* = 89; 13.6%) unit (Fig. 1)

.

**Table 1:** Demographic characteristics of participants

Demographics	N (%)
**Sex**	
Female	361 (46.22)
Male	419 (53.65)
**Total:**	**780 (100)**
**Age (in years) – mean (SD)**	59.53 (19.54)
**Admission Unit**	
Medicine	489 (62.6)
Psychiatry	97 (12.42)
Surgery	194 (24.84)
**Emergency Department visit**	
Yes	655 (83.87)
No	125 (16.01)
**MOST Completion**	
Yes	464 (59.41)
No	316 (40.46)
**MOST Designation**^a^	
M1	37 (4.74)
M2	8 (1.02)
M3	94 (12.04)
C0	34 (4.35)
C1	12 (1.54)
C2	279 (35.72)
**Total:**	**464 (59.41)**
**Provider Completed MOST**	
Physician (MD)	460 (58.9% out of *n* = 780 or 99.1% out of *n* = 464)
Nurse Practitioner (NP)	2 (0.26% out of *n* = 780)
Resident physician (R)	1 (0.13% out of *n* = 780)
Inactive physician (XMD)	1 (0.13% out of *n* = 780)

^a^**MOST Designation** (https://www.interiorhealth.ca/sites/default/files/PDFS/most-orders-for-scope-of-treatment.pdf)

• M1: Supportive care (e.g., symptoms management and comfort measures only)

• M2: Medical treatments within current location of care

• M3: Medical treatments including transfer to higher level of care

• C0: Critical interventions excluding CPR, defibrillation

• C1: Critical care interventions including intubation, but excluding CPR and defibrillation

• C2: Critical care interventions including CPR, defibrillation and/or intubation

### MOST completion

The MOST form was completed for about half of the participants (n = 464; 59.41%) mainly by a physician (*n* = 460; 99.1%). The MOST completion rate was higher through the ED (*n* = 411; 88.58%) than those directly admitted into units/beds (*n* = 53; 11.42%), which represents a statistically significant difference [Pearson χ^2^_(df=2, n=780)_ = 278.67, p < 0.001, φ = 0.598]. Out of 464 individuals with a complete MOST form, 350 (75.4%) persons were admitted in medical, 37 (8%) in psychiatry, and 77 (16.6%) in surgery unit (Fig. 1). Figure 1 illustrates the number of admissions via ED or not as well as admissions per unit with or without a completed MOST form. These findings indicate a statistically significant difference between the patients who completed the MOST form in medical unit compared to ones in surgical and psychiatry [Pearson χ^2^_(df=2, n=780)_ = 79.53, *p* < 0.001, φ = 0.319] with a medium effect size (φ = 0.319). Also, male patients (n = 249; 53.7%) were more likely to complete the MOST form compared to female patients (*n* = 215; 46.3%), but with a non-statistically significant difference [Pearson χ^2^_(df=1, n=464)_ = 0.001, *p* = 0.971].

### MOST designation

#### Level of care by sex

**Fig. 2:**
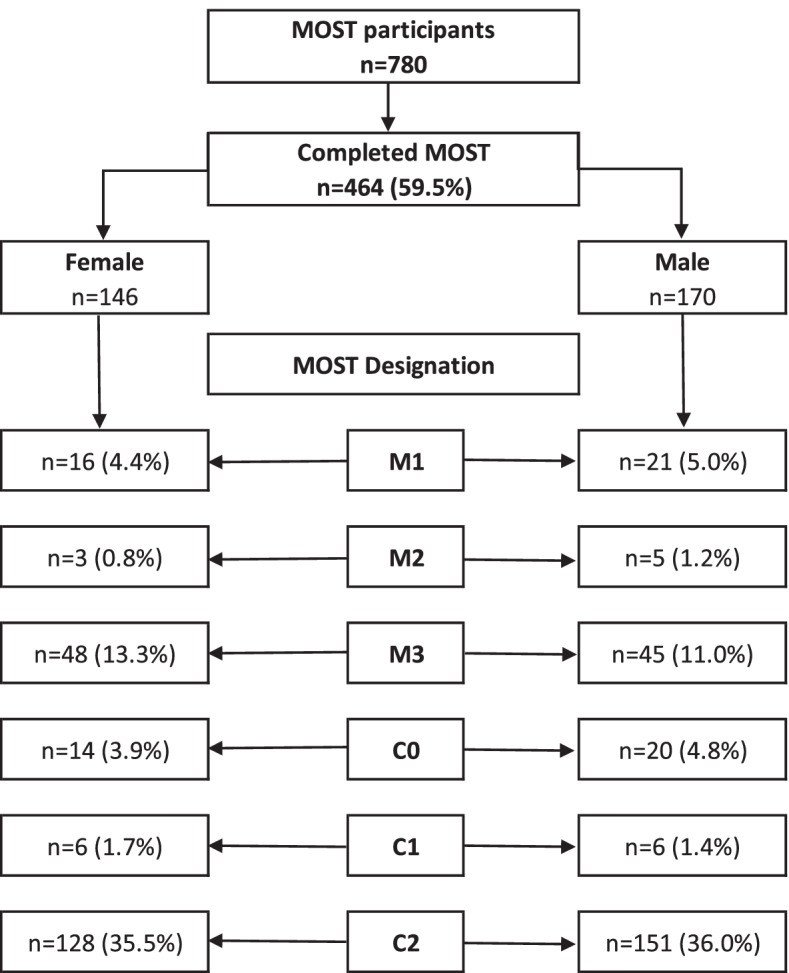
Comparison of care level preferences per gender

The majority of participants with a completed MOST (*n* = 279; 35.72%; 128 females) preferred C2 level of care (i.e., critical care interventions including CPR, defibrillation and/or intubation); 12 (1.54%; 6 females) C1 (i.e., critical care interventions including intubation, but excluding CPR and defibrillation); 34 (4.35%; 14 females) C0 (i.e., critical interventions excluding CPR and defibrillation); 94 (12.04%; 48 females) M3 (i.e., medical treatments including transfer to higher level of care); 8 patients (1.02%; 3 females) M2 (i.e., medical treatments within current location of care); and 37 participants (4.74%; 16 females) M1 level of care (i.e., supportive care such as symptoms management and comfort measures only). Details on participants’ preferences on care level per sex are depicted in Fig. 2. A Pearson chi-squared test regarding the MOST designation preference yielded a non-statistically significant difference between males and females [Pearson χ^2^_(df=6, *n*=780)_ = 1.692, *p* = 0.946].

#### Level of care by unit

In medical unit/beds, most of the study participants preferred C2 level of care (*n* = 188; 38.4%) and their second choice was M3 level of care (*n* = 85; 17.4%). In psychiatry, the majority of participants preferred C2 level of care (*n* = 35; 36.1%) or M3 (*n* = 2; 2.1%). Similarly in surgery unit, the majority of individuals preferred C2 level of care (*n* = 56; 28.9%) or M3/C0 (*n* = 7; 3.6% in either preference). A Pearson chi-squared test yielded a statistically significant difference among admission units in terms of level of care preferences [Pearson χ^2^_(df=12, n=780)_ = 108.295, *p* < 0.001, φ = 0.373].

#### Level of care by sex & unit

Finally, we compared participants’ preferences on level of care per admission unit and by sex. For example, 79 females (35.4%) in medical unit had chosen C2 level of care, and 16 females (7.2%) M1 level of care. On the other hand, all males in psychiatry (*n* = 16; 33.3%) preferred C2 level of care than any other level of care.

### Logistic regression

We performed a direct (standard) logistic regression data analysis to assess the impact of a set of predictors on the odds that the MOST form would be completed. The model contained four independent variables (i.e., sex, age, admission unit, admission date) out of nine in the dataset. The full model containing all predictors was statistically significant (omnibus *χ*^2^ = 207.95, df = 5, *p* < 0.0001; and Hosmer and Lemeshow *χ*^2^ = 2.43, df = 8, *p* = 0.965), indicating that the model was able to distinguish between patients who had a completed versus those who did not complete the MOST form. The model as a whole correctly classified 72.7% of cases.

As shown in Table 2, only two of the independent variables made a statistically significant contribution to the model (i.e., age, admission unit). The strongest predictor of the MOST form completion was age recording an odds ratio of 1.05 (95% CI 1.04 to 1.06), indicating that the odds are 1.05 times greater that older persons would had completed the MOST form than younger ones, controlling for all other factors in the model. The odds ratio of 0.60 and 0.21 for admission in the psychiatry or surgery unit respectively was less than 1, indicating that the odds of the MOST form to be completed decrease by a factor of 0.60 in the psychiatry unit and 0.21 in the surgery unit compared with the medicine unit, all other factors being equal. In other words, patients admitted in the psychiatry or surgery unit had decrease odds of completed the MOST form by a factor of 1.67 (95% CI 1.00 to 2.76) or 4.78 (95% CI 3.26 to 7.04) respectively.

**Table 2:** Logistic regression predicting likelihood of reporting the MOST form completion

Predictors	B	SE	Wald	df	p-value	Odds Ratio (OR)	95% CI for OR	
							**Lower**	**Upper**
Age	.052	.005	105.349	1	.000	1.054	1.043	1.064
Sex	-.035	.169	.044	1	.834	.965	.693	1.344
Admission unit (psychiatry)	-.510	.258	3.904	1	.048	.600	.362	.996
Admission unit (surgery)	-1.567	.197	63.021	1	.000	.209	.142	.307
Admission date	.000	.009	.000	1	.989	1.000	.982	1.018
Constant	-2.136	.356	35.920	1	.000	.118		

## Discussion

The current COVID-19 pandemic, with higher-than-usual rates of death and anxiety among citizens, has challenged the health system by increased levels of care demands and limited human and other resources, and highlighted the need for the general public to engage in ACP, serious illness conversations and wishes regarding sudden or unforeseen health crises including critical care interventions. In a continuously changing context, ACP is significant for people of advanced age or illness (and for others), who wish to make known their preferences in the event of future and/or unexpected changes to health status. In this retrospective study, we aimed to examine the use of MOST form in inpatient units within a BC hospital, to estimate and compare its completion rate, and to inform health policies for continuous, quality and individualized patient care.

### MOST completion

About 72% of study participants admitted through ED in medical beds (63%) primarily. Males were more likely (54%) to complete a MOST form than females, as in another study about integration of the MOST form [8], where 53.5% of participants were females. The MOST form was completed mainly by a physician (99%), but we do not have information about employed NPs in the study site (e.g., how many were fully employed based on their qualifications and scope of practice).

- Frequency

Only about 60% of those admitted to the participating units had completed a MOST form, which is a surprising finding during a pandemic when ACP seems to be highly relevant. Similarly in the Platts-Mills and colleagues’ [21] study, older persons reported having completed a type of ACP document (59%), while only13% of them had either a current code status or any other ACP documentation in their electronic health records. Scholars argue that the uptake of ACP has decreased in some settings [22] due to possible contributing factors that include social distancing and large amounts of information, time pressures, burdens of grief, and complex multi-step ACP processes.

- Unit of admission

Interventions in electronic health records may improve availability, standardization and completion of MOST-like documentation at the point of care [23]. In our study, the completion rate was higher in those persons admitted via ED (89%), which indicates the triage care providers may have been involved in MOST completion and/or had available information about individuals’ preferences and needs at higher rates than other settings as indicated in another study [24]. Our data do not encompass details about person transfers (e.g., from home, residential care), MOST origination and review (prior to or upon admission to ED), or MOST revision during hospitalization on units. Our findings show that persons admitted to medical units/beds were more likely (75%) to have a complete MOST form in comparison with those admitted in surgical or psychiatry beds. At first glance, it may not be surprising that MOST completion rate is lower for those admitted to psychiatric units, although there are important distinctions between mental and physical health considerations for ACP in relation to mental health [25].

- Age

Another findings shows that age was the stronger predictor of the MOST form completion. However, we cautiously interpret this finding because the linearity of the log‐odds was violated for the variable “age” (*p* = 0.025). In an earlier study [8], scholars found statistically significant differences in distribution of age, in the Charlson Comorbidity Index (CCI), in admitting diagnosis and unit. Older persons with a higher CCI were more likely to have a documented MOST. However, it is important to note that recorded preferences of care do not guarantee care concordance [26]. In our study, this kind of data were lacking and we were unable to test concordance of care.

### Study limitations

One of our study limitation is the limited collected data (10 variables only), while the sample size is large (*n* = 780). Another limitation is the limited MOST data from various health organizations due to lack of matching data that could be retrieved via electronic health records. Finally, the limited number of MOST-specific published studies had an impact on the interpretation and discussion of our findings.

### Implications of the study

#### For practice

The study findings have implications for practice. To facilitate and study goal concordant care, designated healthcare providers (i.e., physicians, NPs) need to scholastically and consistently complete the MOST form for every person receiving healthcare the first time they visit a healthcare facility. Since ACP is a patient right to self-determination [27] and a multifaceted family-centered and social process [27], includes both oral discussion and written document for more effective results [28].

#### For education

Implications for education involve all stakeholders. For example, ACP may be hindered due to patient lack of knowledge or lower levels of education [29] and/or physician uncertainty about prognosis [30]. Curricula should include recent and the best available evidence about ACP to prepare healthcare professionals to discuss topics about end-of-life situations with individuals in-person and online [31]. Russell [32] suggested that an important aspect of education pertains to management of emotions from patients, designated decision-makers, and healthcare professionals.

#### For policy

Implications for policy include emphasis on ACP aims and philosophy, implementation and concordance of persons’ wishes and preferences with care perceived that may be misrepresented or misaligned with cultural beliefs about individual autonomy or control [3, 27]. Emphasis on the need for common MOST forms across relevant settings and jurisdictions (e.g., provincial health authorities) and, ideally, across the country is required for continuity of healthcare delivery. Also, policies are needed to facilitate opportunities for all individuals to discuss ACP, record their values and choices, and manage use of limited resources. Finally, it is important the ACP documentation to be included in the electronic health records for easy access to the content with confidence, especially in case of emergency [28].

#### For research

There are plenty implications for future research as we previously discussed. MOST-specific studies are needed focusing on the effectiveness and efficiency of MOST-like forms in facilitating discussions and GoC concordance [4, 32–37] and holistic system of ACP processes [4]. Interventions (e.g., education for all stakeholders) may affect utilization of MOST form [38]. Also, barriers and facilitators to MOST completion need in-depth examination such as social and systemic conditions and other factors related to patients, substitute decision-makers [30] and others. Finally, NPs’ contributions to the MOST completion as well as the input of other healthcare team members such as registered nurses [39–41] and social workers [42] are important areas for research. Finally, evaluation of electronic documentation of the information and standardized tools may support the uptake [43].

## Conclusions

This retrospective study demonstrates that the MOST form is not used as broadly as we expected. Policies need to encourage the development and scholastic use and completion of a common MOST form across provincial health authorities and across the country to promote continuity of care, and raise awareness for making individual decisions about ACP, and determine a decision-maker on potential future health issues. All healthcare providers must be prepared and well-educated to discuss topics relevant to end-of-life with individuals and their families. Also, we suggest further research to illuminate outcomes of systematic use of the MOST form across all jurisdictions. Finally, we emphasize the need for advance electronic systems in place for completion of the MOST form electronically and at the first visit of any care point.

## Data Availability

The dataset used and analyzed during the current study is available from the corresponding author upon reasonable request.

## Abbreviations

ACP: Advance Care Planning
BC: British Columbia
CCI: Charlson Comorbidity Index
CPR: Cardio-Pulmonary Resuscitation
ED: Emergency Department
GoC: Goals-of-Care
ICU: Intensive Care Unit
MOLST: Medical Orders for Life Sustaining Treatment
MOST: Medical Orders for Scope of Treatment
NP: Nurse Practitioner
POLST: Physician or Portable Orders for Life-Sustaining Treatment
TELP: Treatment Escalation/Limitation Plan
